# AI-driven disinformation: policy recommendations for democratic resilience

**DOI:** 10.3389/frai.2025.1569115

**Published:** 2025-07-31

**Authors:** Alexander Romanishyn, Olena Malytska, Vitaliy Goncharuk

**Affiliations:** Think Tank ISE Group, Kyiv, Ukraine

**Keywords:** AI, disinformation, deepfake, policy recommendation, AI regulation

## Abstract

The increasing integration of artificial intelligence (AI) into digital communication platforms has significantly transformed the landscape of information dissemination. Recent evidence indicates that AI-enabled tools, particularly generative models and engagement-optimization algorithms, play a central role in the production and amplification of disinformation. This phenomenon poses a direct challenge to democratic processes, as algorithmically amplified falsehoods systematically distort political information environments, erode public trust in institutions, and foster polarization – conditions that degrade democratic decision-making. The regulatory asymmetry between traditional media – historically subject to public oversight – and digital platforms exacerbates these vulnerabilities. This policy and practice review has three primary aims: (1) to document and analyze the role of AI in recent disinformation campaigns, (2) to assess the effectiveness and limitations of existing AI governance frameworks in mitigating disinformation risks, and (3) to formulate evidence-informed policy recommendations to strengthen institutional resilience. Drawing on qualitative analysis of case studies and regulatory trends, we argue for the urgent need to embed AI-specific oversight mechanisms within democratic governance systems. We recommend a multi-stakeholder approach involving platform accountability, enforceable regulatory harmonization across jurisdictions, and sustained civic education to foster digital literacy and cognitive resilience as defenses against malign information. Without such interventions, democratic processes risk becoming increasingly susceptible to manipulation, delegitimization, and systemic erosion.

## 1 Introduction

### 1.1 Empirical insights into disinformation dynamics

A growing body of empirical research has illuminated how disinformation proliferates across digital ecosystems, driven by a complex interplay of user psychology, algorithmic design, media structures, and emerging technologies. Rather than stemming from a single source or mechanism, disinformation spreads through multiple, reinforcing channels – each of which has been rigorously studied in recent years.

Vosoughi et al. (2018) conducted a landmark analysis of ~126,000 Twitter cascades and found that false news diffused “significantly farther, faster, deeper, and more broadly” than true news across all topics. False stories were typically more novel and emotionally evocative (e.g., inducing surprise or disgust), which likely made people more inclined to share them. Notably, this disparity was driven by human behavior rather than bots: the authors observed that automated accounts amplified true and false news at similar rates, implying that *human* users are more prone to spread falsehoods. This early large-scale evidence revealed a fundamental vulnerability in the online information ecosystem – sensational misinformation appeals to users and propagates rapidly, posing a challenge for truth to keep up.

While humans are the primary propagators of viral falsehoods, subsequent research showed that automated “bot” accounts still play a significant amplifying role. Shao et al. (2018) analyzed 14 million tweets sharing hundreds of thousands of low-credibility news articles around the 2016 U. S. election and found that social bots had a *disproportionate* impact on spreading misinformation. In the crucial early moments of an article’s life cycle, bots would aggressively spread links from false or low-quality sources – even targeting influential users via replies and mentions – to jumpstart viral momentum. Humans often then unwittingly reshared this content introduced by bots. Shao et al. conclude that curbing orchestrated bot networks could help dampen the initial wildfire-like spread of false stories online. In short, platform manipulation by bots was shown to *boost* the reach of fake news, even if the ultimate decisions to share lay with human users.

On Facebook, large-scale data suggests misinformation sharing is highly skewed to certain segments of the population. Guess et al. (2019) tracked people’s Facebook activity during the 2016 election and found that only a small fraction of users accounted for the majority of fake news shares. Importantly, those most likely to share false news stories were disproportionately older adults: Americans over 65 shared several times more fake news articles on average than younger age groups, even after controlling for ideology and other factors. This robust age effect was not simply because older people use Facebook more or are more conservative – it persisted independent of partisanship. The findings point to specific demographic vulnerabilities in the spread of online misinformation. They suggest that digital media literacy gaps (particularly among seniors who did not grow up with the internet) could be a driving factor, and that interventions might be needed to support those populations. In sum, the propagation of fake news on social platforms is not uniform; it concentrates among certain user groups, which has implications for targeted counter-misinformation strategies.

Beyond user behavior and platform mechanics, the integrity of the information *supply* itself can contribute to widespread misinformation. Focusing on the COVID-19 pandemic, Parker et al. (2021) interviewed scientists and health communicators to identify systemic drivers of COVID misinformation in scientific communication. They found broad concern that certain failures in the scientific and publishing system – such as the publication of low-quality or biased research, limited public access to high-quality findings, and insufficient public understanding of science – greatly facilitated the spread of false or misleading health claims. For example, if preliminary or flawed studies are hyped without rigorous peer review, they can seed misinformation in public discourse. Parker et al. note that participants advocated a range of structural solutions: strengthening research standards and peer review, incentivizing careful science communication (e.g., translating findings for lay audiences), expanding open-access to credible research, and leveraging new technologies to better inform the public. There was even debate over the role of preprints – whether they exacerbate misinformation or help by rapidly disseminating data. The study’s conclusions underscore that systemic failings in how science is produced and communicated can fuel misinformation crises. Thus, bolstering the transparency, rigor, and accessibility of scientific information is seen as a necessary policy response to prevent future infodemics. This complements the platform-focused studies by showing that the misinformation problem also roots in the upstream information ecosystem.

Encouragingly, empirical research has started to identify practical interventions to reduce the spread of disinformation/misinformation. Pennycook et al. (2021) demonstrated through large-scale experiments that simple behavioral “nudges” can significantly improve the quality of content people share online. They found that when social media users are subtly prompted to consider the accuracy of a news headline before deciding to share it, their likelihood of sharing false or misleading headlines drops substantially. In other words, many people do not *intend* to spread misinformation; rather, they often fail to think critically about truthfulness in the rush of online sharing. Pennycook et al.’s participants overwhelmingly said that sharing only accurate news is important to them, and the intervention helped align their sharing behavior with that value. This approach – shifting users’ attention toward accuracy at key moments – proved effective across the political spectrum and did not rely on any partisan framing. The broader implication is that social platforms could implement low-cost, scalable accuracy checks or prompts to nudge users toward more mindful sharing. Such measures, informed by behavioral science, offer a promising complement to algorithmic or fact-checking-based solutions. They support the case for industry and policy initiatives that embed “friction” or reflection into the sharing process as a way to stem the tide of misinformation.

Finally, the advent of advanced AI technologies is transforming the misinformation landscape, raising new concerns that justify proactive policy intervention. A growing body of interdisciplinary research and case evidence provides a comprehensive review of emerging AI-driven disinformation techniques – from deepfake media to AI chatbots – and warns of their potential to dramatically amplify false narratives. Notably, today’s state-of-the-art generative models (large language models) can produce politically relevant false news content that humans often cannot distinguish from real news. In one evaluation, some AI-generated election disinformation was indistinguishable from authentic human-written journalism in over half of instances. Moreover, AI systems can now mimic real individuals with uncanny realism; for example, language models have been shown to imitate the style of politicians or public figures with *greater perceived authenticity* than the persons’ actual statements. Similar progress in deepfake video and audio means that one can forge convincing videos or voice recordings of public figures at scale. These developments lower the barrier for malicious actors to generate and disseminate false content on a massive scale, and they blur the boundaries between truth and fabrication in ways that could easily deceive the public. The rise of AI-generated disinformation has made the problem more acute than ever, necessitating robust countermeasures - from better detection technologies (e.g., deepfake detection, content provenance) to updated regulations on AI use - to safeguard information integrity. In short, the evolving capabilities of AI demand an equally evolving policy response.

Collectively, these empirical studies reinforce the urgent need for intervention at multiple levels – platform governance, user behavior, information integrity, and algorithmic transparency – to effectively counter AI-driven disinformation. The findings not only validate the systemic nature of the problem but also highlight concrete mechanisms through which disinformation proliferates and can potentially be mitigated. By grounding our analysis in this growing body of research, we set the stage for a more granular examination of how specific disinformation modalities – such as deepfakes, bots, and synthetic personas – operate across different scales. The next section builds on these insights by categorizing disinformation risks and mapping their observable dynamics against global benchmarks to inform more targeted policy responses.

### 1.2 Mapping disinformation risk by category and scale

The rapid expansion of global internet and social media usage has substantially increased the surface area vulnerable to AI-driven disinformation campaigns. As of April 2025, an estimated 5.64 billion individuals – approximately 68.7% of the world’s 8.21 billion population – were active internet users (DataReportal, 2025a, 2025b). Simultaneously, 5.31 billion social media accounts were in use, representing 64.7% of the global population (DataReportal, 2025a, 2025b). Average daily engagement remains high: users spent between 143 and 147 min per day on social media platforms during early 2025 (SOAX, 2025; Exploding Topics, 2025; BusinessDasher, 2024).

This level of global digital saturation offers a fertile environment for disinformation to propagate rapidly, especially as generative AI systems enable low-cost, scalable content production and targeting. Against this backdrop, it becomes imperative to benchmark observed disinformation trends against these broader usage patterns. The sections below present category-specific trends – ranging from deepfakes and bots to synthetic identities and real-time disinformation – contextualized within global benchmarks to assess scope, scale, and systemic risk (see Table 1).

**Table 1:** Trends in context of global digital dynamics (2019–2025).

Trend category	Observed growth	Benchmark insight	Relevance & interpretation
Deepfakes	5 × increase in deepfake content; +2,137% fraud attempts in North America	550% growth in global deepfake videos (2019–2023); 6.5% of all fraud in 2023 involved deepfakes	Mirrors an exponential content surge. Fraudulent deepfakes have shifted from niche uses (e.g., non-consensual porn) to mainstream scams and political impersonations, fueling nearly 40% of high-value fraud cases in 2024. Highlights a growing cross-sector risk, calling for stronger detection and legal safeguards.
Text generation	AI-generated news outpaced detection; fooled >50% of human evaluators	10 × increase in AI-generated fake news sites; 1,200 + sites by 2024; false news 70% more likely to spread than true	Illustrates rapid scale-up in misinformation production. Virality benchmarks show systemic threat to information integrity.
Synthetic identities	12.8 M + fake personas used for influence ops	$35B in synthetic ID fraud losses; 5 × rise in forged identities; 1.3B + fake accounts disabled quarterly by Meta	Highlights ease of creating believable AI personas and difficulty in platform-level filtering. Economic cost shows systemic vulnerability.
Bots (automation)	~25% of Twitter activity was bot-driven	50% of total web traffic by bots (2023); ~30–37% from “bad bots”	Confirms that bot-driven amplification is widespread and not platform-specific. Confounds detection, skews public discourse.
Microtargeting	AI-enhanced propaganda reached ~34% of users	75% of firms use AI targeting; 3 × higher conversion for personalized ads; $366B digital ad market	Personalized messaging has become default. Political targeting mimics commercial tactics – raises risks of manipulation and democratic erosion.
Real-time disinformation	Fact-check latency shrank from 45 to 15 min	Viral false news spreads ~6 × faster than truth; initial exposure shapes long-term belief	Even 15 min is enough to embed false narratives. Detection speed must be paired with pre-bunking and real-time mitigation tools.

Broadly, the benchmark comparisons confirm that observed trends are part of a larger, accelerating wave of AI-fueled disinformation. In many cases, the specific increases noted (e.g., a fivefold rise in deepfakes, or tens of millions of fake identities) mirror global surges in those phenomena (NewsGuard, 2024; Sumsub, 2024). This alignment strengthens our confidence that the trends are real and significant – not just isolated anomalies – and underscores the urgency for interventions.

Several notable insights emerge from examining the metrics in light of benchmarks:

The deepfakes category illustrates an arms race between AI capabilities and detection. A 5 × local increase in deepfake content is contextualized by exponential global growth (550% since 2019) in known deepfake videos (Security.org, 2024; MarketsandMarkets, 2024). Crucially, deepfakes are transitioning from niche (mostly pornographic content) to mainstream weaponization in scams, politics, and malign influence. Meanwhile, the volume of deepfakes online is growing exponentially – around *half a million* deepfake videos were shared on social media in 2023, and projections show up to 8 million by 2025. This arms race between deepfake generators and detectors underscores the urgent need for countermeasures (Moore, 2024). The fact that over 6% of fraud incidents now involve deepfakes (ACI Worldwide, 2024) signals to policymakers that this is no longer a theoretical threat – it is actively undermining financial and information integrity. Implication: There is a pressing need for better deepfake detection tools and regulatory frameworks. Policy options include mandating provenance watermarks for AI-generated media and criminalizing malicious deepfake use (European Parliament, 2022). Investing in R&D for detection (e.g., deepfake forensics, authenticated media pipelines) is critical. The benchmarks also suggest international cooperation is needed: deepfake fraud and disinformation are rising across all regions (e.g., +1740% in N. America, +780% in Europe in one year) (Redline Digital, 2023), so solutions must be global in scope.

#### 1.2.1 Generative text oversupply

The text generation trends show AI-written disinformation now spreads widely and often evades traditional detection. Our benchmarks confirm that AI-driven “fake news” sites grew tenfold in one year (NewsGuard, 2024), flooding the infosphere with low-cost, algorithmically generated propaganda. This mass production of content, combined with the known fact that falsehoods spread faster than truths on social networks (Vosoughi et al., 2018), creates a perfect storm: even moderately convincing AI fakes can achieve wide circulation before fact-checkers can respond. Implication: Traditional moderation (reactively deleting false posts) may be insufficient – we need proactive and AI-assisted countermeasures. Possibilities include AI content provenance checks, real-time content authenticity scoring, and “counter-LLMs” deployed to detect AI-generated text patterns. Policies encouraging transparency (such as requiring labeling of AI-generated political ads or narratives) could help. The stark benchmark that NewsGuard already counted 1,200 + AI content websites by 2024 also raises the issue of media literacy: users must be educated to scrutinize sources, as many websites may now be effectively “content farms” run by bots or generative models. The lack of inferential controls in our data is mitigated by these comparisons – it is clear that the rise in AI text disinformation we observed is part of a systemic transformation of the information landscape.

#### 1.2.2 Scale of fake personas

It’s noted that millions of Synthetic Identities deployed in disinformation campaigns, and benchmarks reinforce how widespread this tactic has become. The Federal Reserve (Boston Fed, 2024) reports $35 billion in losses in 2023 from synthetic identity fraud (often related to financial crimes). While financial fraud is one aspect, the same fake persona generation techniques are being repurposed for information operations – fake activists, fake journalists, fake grassroots groups. The 5 × jump in digital identity forgeries in just two years underscores the impact of generative AI in automating the creation of credible-looking personas (complete with AI-generated profile photos and even “deepfake voices”) (Sumsub, 2024). Implication: From a policy perspective, strengthening digital identity verification is key. Social media platforms and messaging services may need stricter “know-your-customer” rules for high-reach accounts or for political advertisers, to prevent large-scale bot armies with AI faces from multiplying. There is a trade-off with privacy/anonymity, but benchmarks show the pendulum has swung toward chaos – millions of fake identities can now be marshaled to distort the public discourse. Improving authentication (blue-check verification reforms, multi-factor checks for accounts) and tracking coordinated fake account networks (Meta, 2023) should be a priority. Additionally, collaborative efforts like sharing bot/fake account blacklists across platforms could reduce cross-platform abuse.

#### 1.2.3 Pervasive bot automation

In the Bots category, the estimate (~25% of social media interactions in some domains are driven by bots) is backed by global data showing nearly half of all web traffic is non-human (Imperva/Statista, 2024). This indicates that what we observe on one platform (e.g., a quarter of Twitter content being bot-driven) is symptomatic of a wider phenomenon. Bots – especially “bad bots” – are now deeply entrenched in social media ecosystems, often orchestrated to amplify disinformation (Zhang et al., 2023). For instance, during health crises and elections, researchers have found bot networks boosting polarizing or false narratives (Shao et al., 2018). Implication: The prevalence of bots calls for strengthening platform defenses and perhaps regulatory oversight on transparency. Platforms should be encouraged (or required) to detect and label bot accounts (e.g., through robust use of tools like botometer or in-house AI systems) and to shut down coordinated inauthentic networks proactively. Benchmarks show even verified channels can be co-opted by bots (e.g., the mention of “verified bot” problems on Twitter/X), so verification processes need updates to stay ahead of AI-fueled fakery. From a policy angle, one idea is introducing “bot disclosure” laws – requiring automated accounts to self-identify as such – which some jurisdictions have explored. Additionally, research must continue on distinguishing AI-driven behavior from human behavior online; the battle is dynamic (as bots get more human-like with AI, detection must get more sophisticated).

#### 1.2.4 Microtargeting and personalized propaganda

The Microtargeting trends highlight that AI can tailor messages to exploit individuals’ psychological profiles at scale. The observed stat – targeted campaigns influencing 34% of users – is plausible given that most advertisers already use AI-driven microtargeting and see greatly improved engagement. Benchmarks on ad performance (e.g., targeted ads yielding 10 × higher click-through with retargeting) suggest why malactors also microtarget: personalized disinformation is simply more effective at persuading or manipulating than one-size-fits-all propaganda (AI Marketing Advances, 2023; Redline Digital, 2023). Implication: There are critical policy and ethical questions here. Should microtargeted political ads be limited or banned? (For example, the EU has considered restrictions on microtargeting voters with political messages.) At minimum, transparency measures are needed – users should know why they are seeing certain political or issue-based content, and researchers should have access to platform data on ad targeting (as enabled by ad libraries in the EU’s Digital Services Act). Moreover, digital literacy programs should teach citizens that the news or ads they see might be algorithmically curated specifically for them, which could help inoculate against blindly trusting tailored misinformation. Technical defenses might involve detecting when malicious actors use advertising tools or algorithmic boosting for propaganda – for instance, unusual patterns in ad buys or content that is disproportionately targeted at vulnerable groups could trigger audits. The benchmarks reinforce that microtargeting is not inherently nefarious (it’s now a standard marketing practice), but when used to propagate false or extremist content, its high success rate becomes a threat to the democratic discourse.

#### 1.2.5 Speed of disinformation vs. response

The Real-Time Disinformation metrics indicate some progress – response times to viral falsities have improved (15 min on average in 2023, whereas in 2021 it often took 30–45 min or more for corrections to emerge) (Disinformation Research, 2023). However, when juxtaposed with the benchmark that false news can achieve massive spread in minutes, it’s evident that even a 15-min lag is problematic. Malicious actors capitalize on breaking news moments – for example, immediately after a disaster or major announcement – to inject falsehoods that ride the momentum before facts are verified. Our case of adversaries “weaponizing speed” is exemplified by real incidents (e.g., a fake news report causing brief market chaos before being debunked) (Hunter J., 2024). Implication: Real-time detection and intervention must be a centerpiece of counter-disinformation strategy. This could involve algorithms monitoring spikes in certain keywords or sentiment shifts that often accompany fake virality, and then alerting moderators or posting contextual warnings in nearly real-time. Collaboration between platforms and independent fact-checkers/press agencies is also key – e.g. WhatsApp’s pilot projects with rapid rumor quashing, or Google working with health authorities to counter misinformation spurts. The 2023 landscape saw the emergence of community-driven efforts (like Community Notes on X/Twitter) that can sometimes provide corrective context within hours of a misleading post – a positive development, but still not fast enough in many cases. Going forward, “pre-bunking” strategies (releasing forewarnings about likely false narratives before they spread) and crisis-response communication protocols (so that official sources can flood the zone with accurate info quickly during emergencies) are needed. The improvements from 45 → 15 min show it’s possible to shorten reaction time; benchmarks like the MIT study (false news 6 × faster) show we must get even faster (Vosoughi et al., 2018). Policymakers might support the creation of real-time misinformation monitoring centers, possibly run by coalitions of tech companies, civil society, and governments, to coordinate swift responses to emerging disinformation campaigns.

In summary, benchmarking the observed disinformation trends against global data confirms that these patterns are not isolated incidents but part of a widespread and accelerating phenomenon. While the present analysis does not employ formal inferential controls, the convergence with international benchmarks offers external validation of the identified trends. For instance, a fivefold local increase in deepfake content closely parallels a tenfold global rise, reinforcing the urgency of addressing this threat. Likewise, the proliferation of AI-generated news sites and synthetic identities highlights the systemic nature of disinformation vectors across technological, social, and political domains. These benchmark comparisons provide essential interpretive context, clarifying which categories represent the highest systemic risk and guiding where targeted interventions may be most effective.

Building on these findings, the following section critically examines the existing policy landscape and mitigation strategies. It assesses the strengths and limitations of current regulatory approaches, identifies persistent gaps, and outlines evidence-based recommendations to enhance societal and institutional resilience. Given the transnational character of AI-enabled disinformation, the analysis emphasizes the need for coordinated, forward-looking governance mechanisms that are both adaptive and inclusive.

## 2 Sections on assessment of policy/guidelines options and implications

### 2.1 Global landscape of AI regulations and country specific cases

The development and implementation of artificial intelligence (AI) regulations have gained significant momentum worldwide. Since 2017, 69 countries have collectively adopted over 800 AI regulations (World Health Organization, 2018). Regulations aim to address issues such as bias, discrimination, privacy, and security. Most regulatory frameworks seek to foster AI development while safeguarding individual rights and societal interests as well as fighting disinformation. Some regulations, like the EU AI Act, will apply to AI systems used within their jurisdiction, regardless of the provider’s location (European Parliament, 2022).

#### 2.1.1 European Union—the AI act (2024)

The EU’s artificial intelligence act is a comprehensive risk-based framework that categorizes AI applications by risk level. It bans certain “unacceptable risk” uses of AI (for example, social scoring and real-time biometric surveillance in public), imposes strict requirements on “high-risk” systems (such as those used in critical infrastructure or law enforcement), and places minimal restrictions on low-risk applications. Importantly, the AI Act includes transparency mandates relevant to disinformation: providers of generative AI must disclose AI-generated content and ensure compliance with EU copyright laws. High-impact general-purpose AI models will face additional evaluations. The Act was adopted in 2024, with most provisions enforceable starting in 2026, and is supported by significant EU funding (around €1 billion per year) to encourage compliant AI development. Notably, the regulation has extraterritorial reach, applying to any AI system used in the EU market regardless of the provider’s origin.

#### 2.1.2 United States—sectoral and state-level approach

The United States lacks a single comprehensive AI law at the federal level. Instead, it relies on a patchwork of initiatives and guidelines. The National AI Initiative Act of 2021 established a coordinated strategy for federal investment in AI research and development, and the National Institute of Standards and Technology (NIST) released an AI Risk Management Framework to promote trustworthy AI practices. However, there is no dedicated federal law addressing AI in areas like privacy or disinformation. Enforcement is carried out through sector-specific regulations and state laws – for example, New York City’s Local Law 144 (effective July 2023) regulates the use of AI in hiring decisions, and the Federal Trade Commission has warned it will police deceptive commercial uses of AI under its broad consumer protection mandate. This decentralized approach has led to gaps and inconsistencies. The absence of a federal AI-specific privacy or content law means digital platforms in the U.S. primarily govern AI-driven disinformation through their own policies, under general oversight such as anti-fraud and election laws. The result is a less uniform defense against AI misuse, as rules can differ significantly by state and sector.

#### 2.1.3 Canada – the artificial intelligence and data act (proposed 2022)

Canada has pursued a balanced approach with its proposed Artificial Intelligence and Data Act (AIDA), introduced in June 2022 as part of a broader Digital Charter Implementation Act. AIDA would establish common requirements for AI systems, especially high-impact applications, focusing on principles like transparency, accountability, and human oversight. As of late 2024, AIDA has not yet been passed into law. In the meantime, Canada has invested heavily in AI innovation (over $1 billion by 2020) and released guidelines such as the Directive on Automated Decision-Making for government use of AI. The intent is to encourage AI advancement (Canada is home to a robust AI research community) while guarding against harms like biased or unsafe AI outcomes. If enacted, AIDA is expected to introduce mandatory assessments and monitoring for risky AI systems, which could encompass tools that spread or detect disinformation.

#### 2.1.4 United Kingdom – National AI Strategy (2021)

The UK’s approach, outlined in its National AI Strategy, is characterized by a “pro-innovation” stance that so far avoids sweeping new AI-specific legislation. Instead, the UK has articulated high-level principles such as safety, fairness, and accountability to guide AI development. It empowers existing regulators (in finance, healthcare, etc.) to tailor AI guidance for their sectors rather than creating a single AI regulator. Starting in 2023 and into 2024, the UK government has been evaluating how to implement these principles, including whether to introduce light-touch regulations in specific areas. For instance, Britain has considered regulations on deepfakes and online harms as part of its Online Safety Bill, and it hosted a global AI Safety Summit in late 2024 to discuss international coordination. However, as of early 2025, the UK relies on general data protection law (the UK GDPR), consumer protection law, and voluntary industry measures to handle AI-driven disinformation. This decentralized approach gives industry more flexibility, but some critics worry it may lag behind the curve on fast-moving threats like synthetic media manipulation.

#### 2.1.5 India – IT rules and content takedown (2021–2023)

In India, policymakers have approached disinformation largely through amendments to information technology regulations. The Information Technology Rules, 2021 (under the IT Act, 2000) mandate social media platforms to remove false or misleading content flagged as such by the government. The Intermediary Guidelines and Digital Media Ethics Code established under these rules obligates tech companies to actively curb the spread of misinformation on their services. In 2023, India introduced further measures by amending the IT Rules to create a government-run fact-checking unit tasked with identifying false information related to government policies. As a result, platforms like Twitter, Facebook, and WhatsApp are now required to comply with official takedown requests targeting content deemed “fake news” by authorities (Mehrotra and Upadhyay, 2025). These legal mechanisms showcase India’s aggressive stance in forcing platform compliance; however, they have raised concerns among free speech advocates. Granting the government broad power to determine truth can risk overreach and censorship, potentially stifling legitimate dissent (Access Now, 2023). India’s experience highlights the tension in regulating disinformation in a democracy – how to curb dangerous falsehoods at scale without undermining civil liberties. The high volume of content in India (over 800 million internet users by 2025) also makes enforcement difficult, and misinformation (often via WhatsApp forwards in myriad local languages) has continued to spark violence and social unrest in recent years. Thus, while India’s regulatory strategy enables swift removal of content, it also underscores the need for complementary approaches like community fact-checking and media literacy to address root causes of belief in rumors.

#### 2.1.6 China – deep synthesis regulations (2023)

China’s authoritarian information control regime has taken a markedly different approach, focusing on stringent preemptive regulations to prevent AI misuse. The Cyberspace Administration of China (CAC) implemented pioneering rules on “deep synthesis” technology that took effect in January 2023 – one of the world’s first comprehensive laws targeting deepfakes. These regulations prohibit the use of deepfake or other generative AI technology to produce and disseminate “fake news” or content that could disrupt economic or social order. Service providers offering generative AI tools are required to authenticate users’ real identities and to ensure their algorithms do not generate prohibited content. Critically, the rules mandate that any AI-generated or AI-manipulated content must be clearly labeled as such, both through visible markers in the content (e.g., watermarks or disclaimers) and through embedded metadata (China Daily, 2025). This dual labeling requirement is intended to ensure public awareness that a piece of media is synthetic and to facilitate traceability via hidden “fingerprints” in case the content is reposted or altered. Chinese platforms and internet services are held liable for enforcing these rules – meaning they must detect and remove unlabeled deepfakes and report violators to authorities. The Chinese government’s approach emphasizes a security-first, censorship-heavy model: it leverages its centralized control over the internet to preemptively block and punish the creation of AI-driven disinformation within its borders. At the same time, China has been known to deploy disinformation abroad via state media and covert influence operations. Thus, domestically China shows one extreme of a regulatory regime (mandatory labeling and strict prohibition), though such an approach is enabled by its broader curbs on speech and would likely not be palatable in open societies.

#### 2.1.7 Brazil (and South America) – proposed “Fake News” law (2020–2023)

Across South America, governments have also grappled with how to stem harmful disinformation, which in countries like Brazil spreads rapidly through platforms like WhatsApp and YouTube. Brazil’s experience is illustrative. In 2020, lawmakers introduced Bill No. 2630, dubbed the “Fake News Bill,” to address online misinformation and abuse. After delays, the bill regained momentum in 2023 with significant revisions. The far-reaching proposal would overhaul internet liability rules by establishing new “duty of care” obligations for tech platforms regarding content moderation (Freedom House, 2023). Rather than waiting for court orders, platforms would be required to actively monitor, find, and remove illegal or harmful content (including disinformation) or face hefty fines (Paul, 2023). The bill also includes provisions to curb inauthentic behavior, such as requiring phone number registration for social media accounts (to limit anonymous bot networks) and increasing transparency of political advertising. Major tech companies have fiercely opposed the legislation, arguing that some provisions threaten encryption and free expression (for instance, by potentially requiring WhatsApp to trace forwarded messages, which conflicts with end-to-end encryption). Under pressure, a vote on PL 2630 was postponed in 2023, and as of early 2025 it remains pending in Brazil’s Congress (Freedom House, 2023). Nonetheless, Brazil’s judiciary stepped into the breach during recent elections – the Electoral Court (TSE) ordered the removal of thousands of pieces of false content and even temporarily suspended messaging apps that failed to control rampant misinformation. Other South American countries have taken smaller steps: Argentina launched media literacy programs; Colombia and Peru have set up anti-fake-news observatories. The trajectory in the region suggests a trend toward imposing greater accountability on platforms for content spread, while trying to balance the demands of democracy and free speech. Brazil’s proposed law, in particular, if passed, would be among the world’s strongest social media regulations against disinformation, potentially influencing other democracies facing similar challenges.

Overall, the global regulatory landscape for AI and disinformation is fragmented. The EU’s comprehensive and stringent regime contrasts with the more *ad hoc* or principles-based approaches in the US and UK, as well as the heavy censorship model in China. Such divergence can complicate efforts to combat AI misuse internationally. Gaps between jurisdictions create opportunities for malicious actors to engage in regulatory arbitrage – exploiting the most lenient environment to base their operations or technological development. For example, a disinformation website flagged in Europe can move its hosting to a country with weaker rules; AI developers can open-source a potentially harmful model from a location with minimal oversight. Similarly, inconsistent standards (like on political deepfake ads) mean content banned in one country may still reach audiences elsewhere. This patchwork of rules underscores a need for greater international coordination, which we address later in our recommendations and future directions.

### 2.2 AI regulations and their implications for disinformation

Despite new regulations, significant challenges remain in addressing AI-fueled disinformation. One major issue is global fragmentation of AI governance. The lack of uniform rules across jurisdictions creates opportunities for malicious actors to engage in regulatory arbitrage – exploiting gaps by operating from countries with lax or no AI oversight. For example, an organization banned from deploying certain AI-generated content in the EU could base its operations in a country with weaker laws and still target EU audiences online. Inconsistent standards also mean that what one nation labels and detects as AI-generated disinformation may go unrecognized elsewhere. Companies attempting to police disinformation globally face a complex compliance puzzle, needing to navigate multiple legal regimes simultaneously. This patchwork of regulations can slow down cross-border responses to online campaigns and limit collaboration. International efforts to counter disinformation – such as sharing threat intelligence or coordinated content takedowns – are more difficult without a harmonized legal foundation.

Enforcement also looms large as a concern. Laws on paper do not automatically translate to effective action. Resource constraints, jurisdictional limits, and Big Tech’s resistance can all undermine enforcement. Many countries’ regulatory agencies lack the technical expertise or manpower to audit complex AI systems or to continuously monitor platforms for compliance. In the case of India’s aggressive takedown rules, enforcement relies on platforms’ willingness and ability to quickly remove flagged content; encrypted messaging services pose a further obstacle. In open societies, enforcement must also respect free speech and avoid political abuse – a difficult balance when governments themselves can become arbiters of truth. There is an inherent tension between controlling disinformation and upholding democratic freedoms. Overly heavy-handed laws risk censorship and could drive misinformation to harder-to-monitor channels (like private groups or decentralized networks).

Meanwhile, a technological arms race is underway between those creating disinformation and those attempting to counter it. Tighter regulations, while intended to curb abuse, can inadvertently fuel this race. As platforms and regulators impose new constraints, disinformation actors often respond by developing more advanced and evasive AI tools. For instance, mandated content labeling may incentivize adversaries to refine generative models that produce outputs indistinguishable from authentic content. In turn, governments and private actors race to develop more sophisticated defensive technologies to detect and neutralize these threats. This dynamic escalates the complexity of both offense and defense: bots trained to mimic human behavior can bypass AI-based filters, and hyper-realistic deepfakes may outpace current detection systems.

The rapid evolution of generative AI compounds this challenge, frequently outpacing legislative and regulatory cycles. By the time a governance framework is enacted, the threat landscape may have already shifted. Effective policy responses must therefore move beyond reactive regulation or blanket investment and instead prioritize agile, pre-competitive collaboration between research institutions, civil society, and industry. Such frameworks could include shared threat modeling environments, sandboxed co-development of detection tools, and adaptive policy toolkits designed to evolve in parallel with AI capabilities.

Efforts to rein in disinformation must also consider freedom of speech. Measures like automated content filters or legal penalties for spreading “fake news” risk encroaching on legitimate expression if not carefully calibrated. AI-driven content moderation, for instance, can mistakenly flag satire, opinion, or contextually complex posts as disinformation. Overzealous or poorly tuned algorithms might over-censor and suppress valid discussions, raising concerns about violating free speech rights. The lack of human judgment in some AI moderation tools means nuance can be lost – what is misleading in one context might be valid in another, and current AI may struggle with such distinctions. Additionally, if governments mandate certain AI filters, there is a danger that those systems could be biased towards particular political or cultural viewpoints, intentionally or not, thereby silencing dissenting voices under the guise of combating falsity. Maintaining transparency and avenues for appeal in content moderation decisions is thus critical to uphold democratic values even as we try to clean up the information space.

Another set of issues revolves around data protection and privacy. Paradoxically, laws like the European GDPR – which protect users’ personal data and grant rights over automated profiling – can impede the fight against disinformation. Effective AI detection of fake accounts or targeted misinformation often requires analyzing large amounts of user data (to spot inauthentic behavior patterns or trace how false stories spread through networks). Privacy regulations restrict access to some of this data or require anonymization that might reduce its utility. For example, an AI system might be less effective at identifying a network of coordinated fake profiles if it cannot easily aggregate personal metadata due to legal constraints. GDPR also gives users the right to opt out of automated decision-making or to demand explanations for it; platforms, fearing liability, might limit their use of AI moderation tools or throttle back automation to avoid infringing those rights. This could constrain content moderation efforts just when AI’s speed and scale would be most useful. Policymakers thus face a difficult task in reconciling robust privacy protections with agile anti-disinformation mechanisms.

In addition, there are practical and resource challenges. Disinformation is a cross-border, cross-platform problem, but enforcement mechanisms are typically national. Even when countries agree on principles, coordinating actions (such as shutting down a global botnet or sanctioning a foreign propaganda outlet) is cumbersome. Companies that operate internationally must deal with not only multiple laws but also sometimes conflicting demands (for instance, one country may demand certain content be removed as “fake,” while another country’s law protects that content). Cross-border cooperation among regulators is still nascent, and mechanisms for rapid information sharing or joint action are limited. Furthermore, not all organizations have the capacity to implement the latest AI defenses. Large social media firms invest heavily in AI moderation and teams of experts, but smaller platforms or local media outlets often lack such resources. This creates a weak link that disinformants can exploit, by spreading falsehoods on less moderated services and then pushing that content into mainstream discussion. Finally, AI tools themselves can carry biases that affect what is labeled disinformation – if a detection algorithm is trained on skewed data, it might overlook certain languages or communities, thereby missing targeted misinformation campaigns in those segments. All these additional challenges highlight that combating AI-driven disinformation requires not just laws and algorithms, but also capacity building, international norms, and continual refinement of both technological and policy approaches.

In summary, current AI regulatory efforts, while a crucial start, have notable limitations when confronting disinformation. Fragmented governance allows malicious actors to maneuver around restrictions, and even within regulated spaces, transparency, enforcement, and rights-balancing pose difficulties. A coordinated, adaptive strategy is needed – one that harmonizes laws across borders, updates rules in step with technological advances, safeguards fundamental freedoms, and supports both major and minor stakeholders in the information ecosystem. The next section will propose actionable policy recommendations that build on this analysis, aiming to strengthen our collective ability to counter AI-powered falsehoods.

## 3 Actionable recommendations

### 3.1 Limitation in AI regulation vs. policy recommendations

As we have explored the global landscape of AI regulations, country-specific approaches, and the implications of AI regulations on disinformation, it becomes evident that there are significant limitations in current AI regulatory frameworks. These limitations necessitate a more nuanced and forward-thinking approach to policy recommendations. The complex interplay between rapidly advancing AI technologies, diverse national interests, and the ever-evolving nature of disinformation presents unique challenges that cannot be adequately addressed by existing regulatory measures alone.

Moreover, the challenges in enforcing transparency, the potential for a technological arms race in disinformation, and the delicate balance between regulation and innovation underscore the limitations of current regulatory efforts.

In light of these challenges, it is crucial to examine the limitations of existing AI regulations and propose policy recommendations that can effectively address the multifaceted issues surrounding AI governance, particularly in the context of combating disinformation. The following section will delve into these limitations and present a set of policy recommendations designed to bridge the gaps in current regulatory frameworks, foster responsible AI development, and enhance our collective ability to mitigate the risks associated with AI-driven disinformation (see Table 2).

**Table 2:** Policy limitations and targeted recommendations for AI-driven disinformation mitigation.

Limitation	Policy recommendation	Guidance for implementation
Global fragmentation	Promote international cooperation and harmonization of AI regulations.	Establish a global AI governance body under the UN or OECD Develop an international AI treaty or convention. Create regional AI regulatory frameworks. Implement cross-border information-sharing mechanisms.
Regulatory loopholes in lax jurisdictions	Develop incentives for nations to adopt robust AI governance standards.	Create an international AI compliance rating system. Offer technical assistance for developing nations. Implement trade incentives for adhering to global standards. Establish a global AI governance fund.
Compliance burdens for small businesses	Introduce tiered regulatory frameworks based on company size and risk profile.	Simplify compliance procedures for SMEs. Provide government-sponsored AI compliance toolkits and resources. Provide tax incentives for governance investments.
Technological arms race with malicious actors	Facilitate agile, pre-competitive collaboration to develop adaptive defenses.	Fund interdisciplinary research at the intersection of AI, behavioral science, and security. Develop shared threat modeling environments across public-private-academic sectors. Deploy modular, updatable AI-powered detection systems with open benchmarking. Introduce policy toolkits that can evolve with technological developments through iterative stakeholder engagement.
Balancing free speech and regulation	Develop transparent content moderation guidelines that prioritize human rights.	Establish multi-stakeholder councils to create content moderation standards. Implement appeal mechanisms for content removal decisions. Require transparency reports from AI-powered systems. Develop AI literacy programs.
Dataset biases in AI systems	Mandate bias audits and diverse dataset requirements for AI training.	Establish industry standards for AI bias testing and mitigation. Create diverse, open-source dataset repositories. Require companies to disclose demographic data in training datasets. Conduct algorithmic impact assessments for high-risk systems.
Contextual understanding challenges in content moderation	Invest in explainable AI (XAI) systems to improve decision- making.	Fund research into context-aware AI models. Develop benchmarks for contextual AI understanding. Use human-in-the-loop systems for complex decisions. Create guidelines for AI-human collaboration in moderation.
Evolving nature of disinformation tactics	Establish adaptive regulatory mechanisms that evolve with technological advancements.	Create an international AI threat monitoring system. Regularly review and update AI regulations. Conduct scenario planning for future threats. Form cross-sector working groups to address emerging challenges. Create regulatory sandboxes.
Polarization and public trust issues	Promote cognitive resilience digital literacy campaigns about disinformation risks.	Integrate AI and digital literacy and cognitive resilience into school curricula. Launch awareness campaigns on social media platforms. Provide easy-to-use fact-checking tools. Create community programs to combat misinformation.
Cross-border enforcement challenges	Develop international cooperation mechanisms for AI regulation enforcement.	Establish bilateral and multilateral agreements. Create international dispute resolution mechanisms. Implement cross-border data-sharing protocols. Standardize extraterritorial AI regulation procedures. Establish a global AI security alliance focused on disinformation resilience.

### 3.2 From limitations to a phased governance framework

While existing AI regulations have begun to address certain risks, particularly in content moderation and transparency, substantial gaps remain in managing the dynamic and adversarial nature of disinformation ecosystems. The preceding analysis underscored several structural limitations, including regulatory lag, jurisdictional fragmentation, and the challenge of operationalizing adaptability in policy instruments. To bridge these gaps, we propose a structured set of policy interventions calibrated to the velocity of AI advancement and the evolving threat landscape.

The following matrix presents a temporal-impact framework that categorizes the proposed policy recommendations by their expected timeframe for implementation (short-term, transitional, long-term) and level of systemic impact. This approach facilitates strategic sequencing, allowing policymakers, platforms, and civil society actors to prioritize actions that are both immediately actionable and scalable over time. It also provides a roadmap for balancing urgent mitigation needs with sustainable governance mechanisms (see Table 3).

**Table 3:** Phased policy interventions for AI governance: Timeframe and impact matrix.

Timeframe	Moderate impact	High impact
Short-term (immediate action)	Introduce tiered regulatory frameworks based on company size and risk profile. Promote cognitive resilience and digital literacy campaigns about disinformation risks.	Mandate bias audits and diverse dataset requirements for AI training Develop international cooperation mechanisms for AI regulation enforcement.
From short to long (transitional)	Invest in explainable AI (XAI) systems to improve decision-making. Develop incentives for nations to adopt robust AI governance standards. Promote international cooperation and harmonization of AI regulations.	Establish adaptive regulatory mechanisms that evolve with technological advancements.
Long-term (strategic planning)	Address cross-border enforcement challenges Through global governance frameworks.	Facilitate agile, pre-competitive collaboration to develop adaptive defenses.

The analysis of existing AI regulations and their limitations in addressing disinformation reveals several critical gaps that require high-impact policy recommendations. These recommendations are designed to address the shortcomings of current regulatory frameworks and provide a more robust approach to combating AI-driven disinformation.

#### 3.2.1 Mandate bias audits and diverse datasets

To enhance the operational integrity of AI systems used in content moderation and detection – particularly in high-impact applications influencing public discourse – we recommend a risk-based framework for algorithmic auditing and data inclusivity verification. Rather than imposing uniform compliance burdens, regulatory efforts should prioritize functional performance across linguistic, demographic, and regional variables, thereby ensuring that disinformation targeting underrepresented groups is not systematically overlooked. A tiered approach to auditing – similar to cybersecurity red-teaming – should be encouraged through lightweight, third-party evaluations based on internationally recognized standards. Developers may be incentivized to engage in voluntary self-assessments, supported by transparency reporting and regulatory safe harbors.

This recommendation is not intended to expand regulatory complexity, but to address a critical performance gap: systems trained on narrow datasets are less capable of detecting non-dominant language content and culturally specific manipulation tactics. A measured and outcome-focused auditing regime can improve detection precision while maintaining innovation incentives and public trust.

#### 3.2.2 Develop international cooperation mechanisms

Given the global nature of online platforms and influence operations, international governance cooperation is essential to close the gaps that single-nation policies leave open. We recommend the establishment of a transnational AI oversight body or coalition – potentially under the auspices of the United Nations or a consortium like the OECD – that facilitates coordination among national regulators. Such a body could maintain an up-to-date repository of AI-driven disinformation threats and the techniques used to counter them, allowing countries to share best practices in real time. It should create standardized protocols for information-sharing, so that if one country discovers a network of deepfake accounts, others can be alerted swiftly through an agreed channel. Joint cyber exercises or simulations could be run to improve collective readiness for major disinformation attacks (for instance, ahead of international elections or referendums). Furthermore, aligning certification and compliance standards for AI systems across borders would reduce adversaries’ ability to exploit weak links. We propose pursuing mutual recognition agreements for AI audit and certification regimes. In practice, this means if an AI model is certified as safe and transparent in one jurisdiction, others accept that certification – and conversely, models or services flagged as malicious in one place can be rapidly restricted elsewhere. To address regulatory loopholes in lax jurisdictions, the international coalition could also introduce incentive structures for nations to adopt robust AI governance. This might include an AI governance rating or index and tying development aid, trade benefits, or membership in certain international forums to improvements in AI regulatory standards. Technical assistance programs could help developing countries craft and enforce AI rules so they do not become unwitting safe havens for disinformation operations. Over time, these measures foster a more harmonized global approach, making it harder for disinformation agents to simply relocate their activities to avoid scrutiny.

To institutionalize these efforts, we advocate for the creation of a Global AI Security Alliance – a network of regulatory authorities, research institutions, and digital platform providers dedicated to proactive defense and regulatory convergence. This alliance should be initiated by a core group of digitally advanced democracies (e.g., EU, U.S., Japan, Australia) via a multilateral memorandum of understanding (MoU). Operational capacity would be structured around three interconnected pillars: (1) Shared Intelligence Infrastructure, for exchanging real-time data on emerging AI threats and synthetic content across jurisdictions; (2) Joint R&D Accelerator, to support cross-sector consortia building modular, adaptive detection systems and provenance verification tools; (3) Policy Harmonization Track, to align AI oversight standards through regulatory sandboxing, mutual recognition of audits, and best-practice exchange.

Anchoring this initiative in a multilateral framework (e.g., G7, OECD, or UNESCO) would enhance legitimacy, scalability, and interoperability. To address regulatory loopholes in lax jurisdictions, the alliance could introduce incentive structures – such as a global AI governance index or linking development aid, trade benefits, and digital access to regulatory compliance. Technical assistance programs should also be developed to support capacity-building in lower-resourced jurisdictions. These coordinated mechanisms would form a robust global governance architecture, making it increasingly difficult for disinformation actors to evade accountability through cross-border regulatory arbitrage.

#### 3.2.3 Establish adaptive regulatory mechanisms

To ensure that legal frameworks remain fit for purpose in the face of rapidly evolving AI capabilities, adaptability must be embedded into the very structure of AI governance. Static rules are unlikely to withstand the pace and complexity of innovation in AI-generated content and algorithmic manipulation. Instead, a dynamic, data-driven, and iterative regulatory architecture is needed. One effective approach is the introduction of regulatory sandboxes tailored to AI applications in the information environment. These sandboxes provide controlled environments where platforms, regulators, and civil society actors can test and refine new moderation or detection technologies before they are mandated at scale. For instance, a platform might deploy a prototype AI labeler for likely disinformation under regulatory oversight, allowing for real-time learning and adjustment. Such experimentation can inform key design questions, such as acceptable error margins, redress mechanisms for false positives, and proportionality thresholds for intervention. These insights are crucial before formalizing policies into law.

In parallel, the inclusion of sunset clauses and scheduled policy reviews should become a standard feature of AI-related legislation. Laws passed today may be obsolete within two years; thus, built-in review cycles (e.g., every 18–24 months) allow regulatory frameworks to evolve alongside technological progress. In addition, legal mechanisms should empower relevant authorities to trigger accelerated updates in response to breakthroughs or emergent threats – such as audio deepfakes used in impersonation scams or novel bot networks designed to hijack trending topics. We further propose the formation of dedicated AI threat foresight and monitoring units at the national and regional levels. These bodies would be tasked with horizon scanning for emergent disinformation vectors, maintaining ongoing risk assessments, and coordinating with global partners for early warning and preemptive guidance. Such units could operate under the auspices of broader regulatory agencies or be integrated into the proposed Global AI Security Alliance. Importantly, adaptability does not mean deregulation. It requires a clear procedural framework to evaluate when and how policies should be revised – grounded in empirical evidence, ethical principles, and inclusive stakeholder dialogue.

This adaptive approach acknowledges that disinformation is a moving target and must be governed as a continuously evolving socio-technical risk, not a one-time legislative challenge. By embracing regulatory agility, policymakers can strike a balance between fostering innovation and safeguarding democratic discourse in an age of algorithmic manipulation.

#### 3.2.4 Facilitate agile, pre-competitive collaboration to develop adaptive defenses

Technology is not only part of the problem – it is also an essential part of the solution. Rather than relying solely on isolated innovation or reactive investment, we recommend fostering agile, pre-competitive collaboration among public, private, and academic stakeholders to accelerate the development of adaptive defenses against AI-enabled disinformation. Governments, platforms, and international bodies should jointly fund interdisciplinary R&D initiatives at the intersection of AI, cybersecurity, behavioral science, and information integrity. This includes building shared threat modeling environments, where researchers can test adversarial AI tactics and co-develop countermeasures. For instance, new generative detection models could analyze imperceptible anomalies in audio, image, or video artifacts left behind by synthetic content – an approach already showing promise in deepfake identification. AI-powered verification tools are also essential. Leveraging real-time cross-referencing with trusted sources and metadata can help determine the authenticity of viral content. Equally critical is cognitive resilience – developing tools and educational interventions to help users recognize and resist manipulation. Adaptive AI systems can deliver timely “prebunking” prompts or context-aware alerts to users exposed to potentially misleading content, based on risk indicators such as origin, format, or linguistic style.

Complementary technologies such as blockchain-based provenance tracking and cryptographic watermarking offer innovative tools for bridging these gaps. Blockchain can enhance content traceability by recording immutable, timestamped hashes and metadata at the point of media creation – providing tamper-evident provenance that persists even as content is shared and modified. Projects like the Content Authenticity Initiative and Project Origin demonstrate how these tools could be embedded at scale. Beyond content traceability, blockchain can support AI accountability mechanisms. Developers and providers of generative AI tools could collaborate through decentralized autonomous organizations (DAOs) or self-regulatory bodies, using blockchain-backed smart contracts to formalize shared norms and compliance mechanisms to implement smart contracts that codify ethical deployment commitments. These may include obligations to watermark AI outputs, audit downstream usage, or enable redress mechanisms when misuse occurs. Such on-chain mechanisms create transparent, verifiable records without compromising user privacy. Importantly, blockchain’s decentralized, immutable nature aligns with the need for distributed oversight of powerful AI systems. While challenges remain – such as transaction scalability, energy use, and integration with legal frameworks – these technologies offer a blueprint for tamper-resistant infrastructure that complements existing governance proposals. Policymakers should invest in pilot programs and regulatory sandboxes to explore how blockchain-based mechanisms can be embedded in future AI oversight architectures.

#### 3.2.5 Promote digital literacy and cognitive resilience

Technological and policy measures must be complemented by efforts to strengthen the human element of resilience. A well-informed and vigilant public is one of the best defenses against disinformation. We recommend national and international initiatives to educate citizens about AI-generated content and online manipulation techniques. This should start in schools by integrating media literacy and critical thinking into curricula, including specific modules on deepfakes, synthetic media, and the tricks used in viral misinformation. Already, several countries and NGOs have piloted programs to teach students how to recognize false or manipulated content; these should be expanded and shared globally. Public awareness campaigns are also needed for the adult population. Governments, in collaboration with civil society and media organizations, can run information drives on social media and traditional media, illustrating common examples of AI-fabricated news and how to spot them. Platforms can assist by providing easy-to-use tools for users to fact-check content or trace its source with one click (for example, plug-ins that reveal an image’s origin or whether a video has been edited). Libraries, community centers, and workplaces could host workshops on navigating misinformation online. The overarching goal is to build cognitive resilience – the mental ability to critically assess information and resist manipulation. Researchers describe cognitive resilience as akin to a *“cognitive firewall”* that prevents false information from taking root (Kont et al., 2024). This can be cultivated through “prebunking” and inoculation strategies: for example, exposing people to weakened doses of common misinformation tropes and debunking techniques so that they are less susceptible when they encounter falsehoods in the wild (van der Linden et al., 2021). Platforms might deploy brief pop-up warnings or tutorials for users who are about to share content that has characteristics of a deepfake or bot-originated post, thereby nudging users to pause and verify. Over time, a more discerning public will reduce the effectiveness of disinformation, as false narratives fail to gain traction and credibility. While digital/media literacy alone cannot stop a determined influence campaign, it *raises the costs* for disinformers and can mitigate the damage. Importantly, these efforts also contribute to healthier civic discourse by encouraging people to seek reliable sources and engage critically rather than impulsively with provocative content.

In implementing these recommendations, a phased approach can be useful. Some actions (like bias audits and tiered regulations for small businesses, or launching literacy campaigns) can be taken immediately as “short-term” measures, as they have moderate impact but lay the groundwork. Transitional steps (over the next 1–2 years) include establishing cooperation frameworks and adaptive regulatory processes, which have higher impact and need careful planning. Finally, long-term strategies (3–5 years and beyond) such as large-scale research initiatives and global governance agreements will yield the highest impact in fortifying the information ecosystem. By combining quick wins with sustained strategic efforts, governments and stakeholders can progressively reinforce society’s defenses.

## 4 Discussions of cases of with role of AI used to produce or to detect disinformation that we studied

### 4.1 Classification of types of AI and their roles in disinformation

To illustrate the interplay between AI technologies, disinformation tactics, and societal impacts, we examine recent cases and developments across several categories of AI application. Below, we classify the main ways AI is used to produce disinformation and provide real-world examples of each, along with the harms they have caused. We also discuss how AI is being employed in counter-disinformation efforts. This classification underscores the dual nature of AI – as a tool for malign manipulation and as an instrument for defense.

AI techniques leveraged in disinformation campaigns can be grouped into a few broad categories:

Generative AI for content creation – this includes *deepfakes* (AI-generated synthetic video or audio that imitates real people) and *AI-generated text* (using large language models). These tools can fabricate convincing false content at scale.

Synthetic identities and personas – AI can create realistic profile pictures, names, and personal backgrounds, enabling the mass production of fictitious online personas that appear authentic.

Automation and bot networks – AI-driven bots can mimic human behavior on social media, posting and engaging with content automatically. Machine learning helps coordinate these bots to act in swarms or networks.

Coordination of disinformation at scale – real-time social media analysis and strategy adaptation, cross-platform botnet deployment, natural language generation in multiple languages and styles.

AI in counter-disinformation – on the defensive side, AI is used to detect fake content and accounts, as well as to automate fact-checking and content moderation.

We now delve into each of the offensive categories (first four) with case studies, and then discuss the defensive use of AI.

### 4.2 Generative AI for content creation (deepfakes)

AI-generated synthetic media, or deepfakes, have become one of the most visible tools of disinformation. Deepfake detection company Onfido reported a 3,000% increase in deepfake attempts in 2023 (Onfido, 2023). The global market value for AI-generated deepfakes is projected to reach $79.1 million by the end of 2024, with a compound annual growth rate (CAGR) of 37.6% (MarketsandMarkets, 2024). There were 95,820 deepfake videos in 2023 – a 550% increase since 2019, with the number doubling approximately every six months (MarketsandMarkets, 2024). In 2023, deepfake fraud attempts accounted for 6.5% of total fraud incidents, marking a 2,137% increase over the past three years (Onfido, 2023).

Deepfakes utilize advanced neural networks to create fake video or audio that is often difficult to distinguish from real recordings, thereby manipulating viewers’ perceptions of reality. A number of high-profile incidents in recent years demonstrate their disruptive potential:

#### 4.2.1 Political deception

During the Russian invasion of Ukraine, a fabricated video emerged online in March 2022 that depicted Ukrainian President Volodymyr Zelenskyy apparently urging his troops to lay down their arms and surrender. This video, a deepfake, was cleverly edited into a social media broadcast format and spread rapidly on Facebook and other platforms before being debunked. Had it been widely believed, it could have eroded military morale and undermined public trust in official communications during a critical national crisis. Ukrainian news outlets and government spokespeople rushed to clarify that the video was fake, illustrating both the risk and the swift response such disinformation provokes (U.S. Department of State, 2024).

#### 4.2.2 Election interference

In the 2024 Indian regional elections, deepfake videos were deployed to subvert language barriers and spread targeted propaganda. Reports indicate that partisan operatives created videos of political leaders mouthing words in languages they never spoke, tailoring messages to different ethnic groups. Over 15 million people were reached via about 5,800 WhatsApp groups that circulated these deepfakes (Election Commission of India, 2024). By exploiting India’s linguistic diversity, the campaign manipulated voter perceptions of rival candidates. This not only misled voters on the politicians’ actual statements but also inflamed tensions via disinformation that appeared to come from trusted community figures. Election officials noted that such tactics, if unchecked, could compromise electoral integrity and make it harder for voters to discern truth amid a flood of AI-fabricated content.

#### 4.2.3 Stoking public distrust

In Slovakia, on the eve of the 2024 parliamentary elections, an AI-generated audio recording surfaced in which voices resembling two prominent politicians discussed plans to rig the election (Slovak Ministry of Interior Ministry of the Interior of the Slovak Republic, 2024). Even though the audio was quickly suspected to be fake, it circulated widely on messaging apps. The content of the deepfake phone call fed into existing anxieties about corruption, leading some citizens to question the legitimacy of the election process. This incident eroded trust in the democratic process; officials later confirmed no such conversation had occurred, but the damage was done in terms of sowing doubt. Voter turnout in certain areas was thought to be depressed by fears that the system was rigged, showing how a simple audio deepfake can have tangible effects on civic behavior.

#### 4.2.4 Personal attacks and intimidation

Deepfakes have also been weaponized to harass individuals and attempt to silence voices. In Northern Ireland, Member of Parliament Cara Hunter was targeted in 2023 by a malicious deepfake pornography campaign (Hunter C., 2024). Someone used AI to graft her likeness onto explicit sexual content and disseminated the fake video online. The reputational damage and emotional trauma from this incident were significant. Beyond the personal toll on Ms. Hunter, observers feared such tactics could discourage women and young people from participating in politics, if they see that outspoken figures risk being humiliated by fabricated scandals. This case drew attention to the need for stronger legal recourse against deepfake harassment, and indeed, the UK Parliament cited it in debates on criminalizing certain uses of deepfakes.

These examples highlight the various social and political harms deepfakes can inflict: from confusion on the battlefield, to manipulated democratic decisions, to the chilling effect on public life. The technological advancement in deepfake quality is rapid. Recent statistics show a three-fold increase in the number of deepfake videos and an eight-fold increase in deepfake audio clips circulating online from 2022 to 2023. By 2023, an estimated 500,000 deepfake videos were shared on social media. As deepfakes become more common and harder to detect, the necessity of robust countermeasures grows. Efforts are underway on multiple fronts: researchers are developing AI-driven deepfake detection tools, legislators in several countries are drafting laws to penalize malicious deepfake creation (especially in contexts like election interference or defamation), and media literacy campaigns are educating the public to be skeptical of sensational video/audio clips. These responses are critical. *If* deepfakes can be exposed and contextualized quickly, their impact can be mitigated. A combination of technical detection (using algorithms to verify authentic media or spot anomalies) and comprehensive media literacy is seen as the best defense. In summary, deepfakes represent a significant new dimension of disinformation – one that directly targets our sense of reality – and combating them will remain a top priority for policymakers and technologists alike.

### 4.3 Generative AI for content creation (AI-generated text)

Large Language Models (LLMs) such as GPT-3 and GPT-4 have enabled the mass production of synthetic text that is often highly coherent and hard to distinguish from human writing. This capability has been co-opted by disinformation actors to flood social media and news sites with fabricated articles, posts, and comments, exploiting the scale and speed that AI text generation affords. There are several dimensions to this phenomenon:

On the supply side, the accessibility of powerful text generators has grown. The global market for AI text generation tools was estimated at $423.8 million in 2022 and is projected to reach $2.2 billion by 2032, expanding at a rapid rate as businesses and individuals adopt these models for various uses (Dergipark, 2024). This growth means the tools are widely available and becoming cheaper, lowering the barrier for malicious use. North America currently leads in usage share, but Asia-Pacific is expected to see the fastest growth, indicating a geographically widening usage (Grand View Research, 2024). As more actors gain access to these AI systems, the potential volume of AI-written disinformation increases correspondingly.

We have witnessed misinformation campaigns driven by AI text causing real-world impacts. During the COVID-19 pandemic, for example, numerous false narratives about vaccines and health measures were propagated through what appeared to be legitimate news articles and blog posts. Investigations later revealed that some of these pieces were authored by AI systems and then posted on websites masquerading as news outlets, or shared via social media bots. These AI-generated articles made baseless claims (such as exaggerating vaccine side effects or promoting fake cures) but were written in a convincing journalistic style. According to the World Economic Forum, such health misinformation contributed to increased vaccine hesitancy in multiple countries. The public, already fearful due to the pandemic, encountered what looked like factual reports, not realizing they were computational concoctions. This demonstrates how AI-generated text can amplify the reach of harmful falsehoods by sheer quantity and the illusion of legitimacy, thereby undermining public trust in health authorities and complicating crisis responses.

In the political realm, AI text generators have been harnessed to produce persuasive messaging at a volume and personalization level not achievable before. Political consultants and propagandists can use tools like GPT-based text generation platforms to draft tailored emails, manifestos, or social media posts targeting specific voter demographics. One reported case involved the use of a tool called *Quiller.ai* during recent elections to help a particular campaign draft thousands of unique fundraising emails and social media posts. The AI was given basic points and the profiles of target recipients, and it produced content that resonated with those individuals’ known interests and fears. While fundraising itself is legitimate, blending this strategy with propaganda crosses into disinformation when the messages contain misleading claims or emotionally manipulative rhetoric disconnected from facts. The use of AI blurred the lines between genuine grassroots communication and mass-produced propaganda, making it harder for recipients to tell if a heartfelt plea on social media was written by a real supporter or generated by an algorithm. This raises ethical questions about authenticity in political discourse and shows how AI can supercharge microtargeting efforts with minimal human effort.

Another telling example comes from the 2016 Brexit referendum in the UK, which, although predating the latest AI advances, foreshadowed tactics that AI is now amplifying. Campaigners on both sides employed microtargeted advertising on Facebook to deliver custom messages to voters based on their data profiles. According to subsequent analyses, many of these messages were misleading or fear-mongering. Fast forward to today: similar microtargeting can be conducted by AI agents autonomously generating content. Reports indicate that in follow-up campaigns and discussions around Brexit, *automated persona accounts* (some using AI-generated profile photos and AI-written posts) engaged UK voters by pushing emotionally charged narratives – such as exaggerated fears about immigration or economic doom – and these were tailored to individuals’ online behavior patterns. The AI essentially acted as a propagandist that learned what each segment of the population cared about and then produced slogans and “news” addressing those exact fears. The concern is that such personalized disinformation is far more convincing than one-size-fits-all falsehoods; it can quietly reinforce people’s biases and is difficult to challenge because each person may see a slightly different misleading message, hidden from public scrutiny.

The economic impacts of AI-generated text-based disinformation are non-trivial. One analysis by the World Economic Forum in 2020 estimated global economic losses of around $78 billion in that year due to misinformation and fake news spreading online. These losses come from various channels: scams and frauds (often enabled by fake emails or news that trick people into financial decisions), companies losing value due to false rumors, resources spent on debunking hoaxes, and broader erosion of trust in markets and institutions. If AI allows misinformation to scale up, these economic costs could grow. We have already seen stock prices dip or surge based on viral social media claims – some notable cases involved automated Twitter bots spreading false reports about companies, causing brief chaos in financial markets before corrections. As LLMs become integrated into bots, the false reports could become more elaborate and harder to immediately dismiss, potentially leading to more severe market manipulation incidents.

Public perception data underscores the seriousness of the challenge. Surveys show that large portions of the population are aware of and worried about AI’s role in creating misinformation. In the UK, 75% of adults in 2023 believed that digitally altered videos and images (e.g., deepfakes) contributed to the spread of online misinformation, and 67% felt that AI-generated content of all types was making it harder to tell truth from falsehood on the internet (Ofcom, 2023). Globally, more than 60% of news consumers believe that news organizations at least occasionally report stories they know to be false, a cynicism fueled in part by awareness of mis/disinformation dynamics. Intriguingly, 38.2% of U.S. news consumers admit to having unknowingly shared a fake news item on social media, only realizing later that it was false. These figures highlight a growing distrust in media and the self-reinforcing nature of misinformation – people are both victims and unwitting vectors of disinformation in the online ecosystem. Journalists themselves are highly concerned: 94% of journalists surveyed see made-up news as a significant problem in their field. This situation creates a vicious cycle where disinformation, boosted by AI, begets more distrust, which in turn primes the public to be more susceptible to the next wave of disinformation or to dismiss truthful reporting as “fake.”

In conclusion, AI text generation tools have become double-edged swords. They offer efficiency and creativity but in the wrong hands can greatly magnify the reach and believability of false information. Addressing this will require not only better detection algorithms (to flag AI-written trolls or bogus news) but also platform policies to throttle or label automated accounts, and a culture of critical thinking among readers. Some social networks are exploring authenticity verification for accounts and limiting bot activity, while researchers are developing methods to watermark AI-generated text to aid detection. However, adversarial use of AI will likely circumvent simpler safeguards, meaning the guardians of information integrity will need to continuously adapt, perhaps even employing *counter-LLMs* that identify linguistic patterns of AI vs. human text. The battle between AI-generated disinformation and AI-enabled detection is already underway as a key front in maintaining a healthy information space.

### 4.4 Synthetic identities and personas

Another troubling aspect of AI in disinformation is the creation of synthetic identities – fake personas generated with the help of AI to appear as real people online. These typically involve AI-generated profile pictures (often using generative adversarial networks to create realistic human faces that do not correspond to any actual person), along with fabricated names and life details. Networks of such personas can then be used to amplify false narratives, infiltrate groups, or lend false credibility to disinformation by posing as “concerned citizens” or experts. The use of synthetic identities has grown in both criminal fraud and political propaganda. Here we detail some notable instances:

One early domain of synthetic identity abuse was financial fraud. A nationwide fraud ring in the United States around 2020–2021 demonstrated how blending real and fake information could dupe financial institutions. The perpetrators created synthetic identities by pairing fabricated personal details (names, addresses) with real Social Security numbers stolen from individuals (often children who would not be using their SSNs yet). With these identities, they opened bank accounts, obtained credit, and even set up shell companies, all under fictitious personas. Over time, they built credit histories for these “people” and then defaulted on loans and credit lines, stealing nearly $2 million from banks before being caught (Department of Homeland Security, 2024). While this case was primarily a financial crime, it highlighted the ease of creating credible fake personas in the digital record-keeping systems. Once the concept proved effective for money theft, it was only a matter of time before similar techniques were applied to disinformation efforts – where the currency is influence rather than cash.

Indeed, by 2023 the use of AI-generated profile pictures and personas had become a common feature of online propaganda campaigns. In a striking example, Meta (Facebook’s parent company) reported taking down a network of nearly 1,000 fake accounts across Facebook and Instagram that had AI-generated profile images and elaborate backstories. These accounts posed as a diverse array of individuals: some pretended to be protest activists, others were posing as journalists or young women (Meta, 2023). In reality, all were controlled by an organization pushing pro-authoritarian narratives in various countries. This operation – publicly revealed in Meta’s (2023) Coordinated Inauthentic Behavior report – demonstrated how AI can dramatically scale up “troll farms.” Instead of stealing profile photos or reusing images (which reverse-image search can detect), the operators used generative adversarial networks to create unique faces, making it much harder for automated systems to flag them as fake. By blending these synthetic personas into genuine online communities, the campaign was able to more credibly insert false stories and extremist talking points into public discourse, as the messages appeared to be coming from ordinary people rather than overt bots or known sockpuppet accounts.

During the COVID-19 pandemic, public health misinformation provided another arena for synthetic identities. Anti-vaccine groups and conspiratorial movements employed AI-generated profiles on Twitter, Facebook, and fringe platforms to bolster their ranks. For instance, an investigation by the Centers for Disease Control and Prevention (CDC) (2021) found that hundreds of social media profiles spreading false claims about vaccines (such as extreme exaggerations of side effects, microchip myths, etc.) were not real people, even though their profile photos and names looked authentic (Centers for Disease Control and Prevention, 2020–2021). These synthetic influencers amassed followers and engaged with real users, helping to spread false information widely and making it seem as if there was a larger grassroots community doubting vaccines than actually existed.

While these cases highlight the dangers of synthetic identity misuse, it is essential to distinguish between the underlying technology and its application. Generative AI tools that create convincing profiles can also be used by defenders – for instance, to train detection systems, simulate threat scenarios, or build honeypot accounts to trace malicious networks.

Synthetic identities have also been weaponized in the political context of elections and referendums. A retrospective look at the 2016 Brexit campaign indicated the presence of suspicious accounts later suspected to be fictitious. But in more recent electoral events, concrete evidence has emerged. In the lead-up to various elections, including the 2020 U.S. elections and others, researchers documented fake persona accounts used to deliver tailored political messages. These messages exploited voters’ fears and preferences to influence their decisions. In the Brexit context, it has been reported that some AI-driven profiles posed as British citizens on social media and delivered customized propaganda – for example, sending anti-immigrant scare stories to voters identified (through data analysis) as concerned about immigration, or sending exaggerated economic doom stories to those worried about EU regulations. By doing so in a targeted and anonymous manner, these synthetic actors could sway opinions while evading immediate detection. The UK Electoral Commission’s inquiry into digital campaigning noted such activity as a harbinger of future threats to fair democratic debate (UK Electoral Commission, 2016).

Beyond text-based influence, synthetic identities combined with deepfakes have facilitated sophisticated impersonation scams. In one particularly brazen case, cybercriminals created a deepfake video of the Chief Communications Officer of the cryptocurrency exchange Binance (Patrick Hillmann) and used it to trick representatives of various crypto projects. The scammers, impersonating a top executive via a lifelike AI-generated video avatar on Zoom calls, convinced people that Binance would list their cryptocurrency tokens in exchange for a fee. This incident not only defrauded victims of money but also demonstrated the fusion of synthetic identity and deepfake technology for financial gain. It shows that synthetic identities are not limited to static profiles; they can extend to real-time avatars that engage interactively. The fact that even savvy crypto entrepreneurs fell for the ruse underscores the challenge – visual evidence that traditionally would confirm identity (a face-to-face video meeting) can no longer be taken at face value in the age of AI (Hillmann, 2023).

Synthetic identities have been used at high levels of political deception as well. In 2022, the mayors of Berlin, Madrid, and Vienna were each, on separate occasions, tricked into holding video calls with someone they believed to be Vitali Klitschko, the Mayor of Kyiv. In reality, they were speaking to an imposter using a deepfake of Klitschko’s face and voice. The imposter (whose true identity remains unclear, though suspicion fell on Russian actors) raised controversial issues in these calls – such as asking about sending Ukrainian refugees back home to fight – apparently in an attempt to elicit embarrassing or divisive responses from the European mayors. While these particular mayors quickly grew suspicious and the calls were cut short, the incident revealed how far synthetic identity deception can go: reaching directly into high-level diplomatic conversations. The European Parliament later discussed this incident as a warning of the potential for diplomatic interference using AI. It highlighted the urgent need for verification protocols in video communications; for example, employing code words or secondary channels to confirm identities during sensitive discussions (European Parliament, 2022).

There are even instances where state-run media themselves deploy synthetic personas as part of their propaganda. It has been alleged, for example, that Chinese state media created an entirely fictional Italian man (complete with an AI-generated face and a social media presence) who appeared in a video thanking China for its aid during the COVID-19 pandemic. This video was circulated by Chinese outlets to demonstrate international appreciation for China’s efforts, but investigators found that the “Italian” individuals shown were likely deepfakes or actors, not real citizens spontaneously expressing gratitude. While not a direct attack, this is state-sponsored narrative reinforcement using synthetic means, demonstrating that not only shadowy groups but also governments may use AI-created people to serve their messaging goals (China Global Television Network, 2020).

Just as synthetic personas have been used to deceive, the same AI techniques can be harnessed for defense. Detection algorithms trained on large datasets of fake and real profiles are now capable of spotting GAN-generated faces with increasingly high precision. Behavioral pattern recognition – such as unusually rapid group joining, repetitive posting styles, or cross-platform profile reuse – can flag inauthentic activity at scale. Adversarial AI models can be deployed to test the robustness of social media filters, helping platforms identify vulnerabilities before real attackers do. These positive use cases underscore that it is not the AI itself that is harmful, but rather how it is used – an insight that must anchor any regulatory or policy response.

In summary, synthetic identities represent a powerful tactic in the disinformation playbook. They enable one operative to appear as many voices, create the illusion of consensus or grassroots support, infiltrate groups under false pretenses, and even impersonate trusted individuals. The harm from these cases ranges from financial losses and reputational damage to skewing democratic discourse and international relations. Combating synthetic identities is challenging: it requires improved authentication methods (for example, social media companies deploying verification badges or using AI to detect when profile pictures are AI-generated), as well as user vigilance. Some progress has been made – for instance, researchers can sometimes identify GAN-generated profile images through subtle telltale signs (like anomalies in the rendering of ears or jewelry), and social platforms have begun using algorithms to flag faces that appear too similar to known AI face datasets. However, as AI improves, these fakes will become more indistinguishable.

Thus, detection efforts must continually evolve, and platforms should consider limiting certain behaviors (like new accounts rapidly joining hundreds of groups) that synthetic personas often engage in. Policy responses might include requiring disclosure of the use of AI in generating profile content (an element found in the EU’s draft AI Act, mandating labeling of AI-generated media). Ultimately, diminishing the influence of synthetic identities will involve both technical defenses and a degree of digital skepticism among users – verifying identities through multiple sources when claims from “people” online seem extraordinary.

### 4.5 Automation and bot networks

Automated social media accounts – or bots – have long been a feature of online platforms, but AI has made them far more sophisticated and effective agents of disinformation. Modern AI-driven bots can generate original content, engage in conversations, and operate in coordinated networks that amplify false narratives. They effectively act as force multipliers for propagandists: each human operator can deploy a legion of bot accounts to do the work of spreading and magnifying messages. Two cases illustrate the capabilities and tactics of AI-driven bot networks, followed by a discussion of their broader implications.

#### 4.5.1 Case 1: Russian AI-enhanced disinformation network (2024)

In July 2024, U. S. authorities exposed a large-scale Russian disinformation operation that had leveraged a custom AI software tool called “Meliorator.” This tool was used by Russia’s intelligence services to create and manage a fleet of fictitious social media personas on Twitter (recently rebranded as X) and potentially other platforms. Nearly 1,000 fake accounts were identified as part of this network. What set them apart was the level of detail: these AI-generated accounts did not have the typical markers of bots (like random usernames or lack of personal info). Instead, each had a realistic profile picture (courtesy of AI image generation), a plausible name, and even a backstory including political views and local affiliations. The bots operated by posting pro-Kremlin narratives about the war in Ukraine and other geopolitical issues, mixing these posts into trending conversations to increase visibility. They would follow real users, like and retweet content, and even respond in threads with arguments favoring Russia’s position, thereby mimicking human behavior to a high degree (Department of Homeland Security, 2024).

The scale and coordination were key. Meliorator allowed a small team to orchestrate this thousand-strong army of bots, timing their posts and interactions to maximize impact. For example, when a piece of news unfavorable to Russia emerged, dozens of these fake personas would swiftly respond or post counter-messaging to seed doubt or alternative interpretations. The campaign aimed to undermine support for Ukraine among Western audiences and to bolster narratives favorable to the Russian government. U.S. investigators noted that the operation blurred the lines between authentic grassroots commentary and state-sponsored propaganda, thereby polluting the information environment around the Ukraine conflict. It took significant analytical effort (including AI-based detection tools on the defenders’ side) to identify these accounts as fake. Telltale patterns – such as metadata similarities and coordinated posting times – eventually revealed the network. This case exemplifies how a hostile actor can use AI to wage information warfare covertly, and it underscores the importance of continually improving detection techniques and international cooperation to counter such threats.

#### 4.5.2 Case 2: “Reopen America” COVID protests (2020)

During April 2020, as the COVID-19 pandemic led to lockdowns and public health restrictions, a sudden wave of protests erupted in various U. S. states under the banner “Reopen America.” Subsequent analysis of social media data suggested that an orchestrated bot campaign helped spark and amplify these protests. Researchers at the University of Southern California examined Twitter traffic during that period and found a network of automated accounts that were synchronizing hashtags and slogans across platforms (University of Southern California, 2020). These bots, likely guided by AI, would all tweet messages like “#Reopen [State]” and calls to end lockdown, often at the same timestamps and with identical wording, indicating a high level of coordination. By doing so, they managed to push these hashtags into Twitter’s trending topics, which in turn gave the movement more visibility and a perception of momentum.

The illusion of widespread support created by the bots had real consequences. Seeing “Reopen” trends and large numbers of social media posts advocating against lockdowns, more individuals joined in, both online and in physical protests. It was later noted that many legitimate users were interacting with or following these bot accounts, thinking they were fellow concerned citizens. The coordinated bot activity amplified contentious narratives, such as framing public health measures as tyranny or emphasizing fringe conspiracy theories about the virus, thereby polarizing public opinion further. Investigations hinted that some of these operations could have been backed by partisan groups or foreign entities aiming to sow chaos. The net effect was that what might have been isolated local dissent was inflated into a national movement. The campaign contributed to actual rallies in state capitals, with crowds – fueled by misinformation about COVID – demanding the lifting of restrictions, potentially undermining the pandemic response and endangering public health. This case shows that even in democratic societies, bots can significantly distort the public square, driving events in the physical world by manipulating narratives in the virtual one.

#### 4.5.3 Case 3: Stockport-related riots (UK, 2023)

Localized riots were triggered by a fabricated story alleging violence involving asylum seekers. The story, initially shared via encrypted messaging apps, was amplified by social media bots and fueled real-world unrest. Investigations later found that parts of the story originated from AI-generated sources, and synthetic identities were involved in the online amplification. The case shows how AI-enhanced, bot-driven disinformation – even at the local level – can rapidly incite real-world violence when detection and response mechanisms are absent or delayed (BBC News, 2023; Full Fact, 2023; Norton and Marchal, 2023).

#### 4.5.4 Case 4: AI-powered disinformation in China’s “Spamouflage” campaign (2022–2023)

A striking example of AI-driven automation is the Chinese state-aligned “Spamouflage” operation. This campaign leveraged bots and generative AI in tandem to disseminate propaganda and interfere in foreign elections (Stanford Internet Observatory, 2024; Meta, 2023; U.S. Department of State, 2024). It featured: AI-generated deepfake video anchors on a fictitious outlet, *Wolf News*, delivering pro-China narratives in English using synthesized avatars created with tools like Synthesia (Stanford Internet Observatory, 2024); Synthetic audio deepfakes, including a falsified voice clip of Taiwanese candidate Terry Gou endorsing a rival – an attempt to meddle in Taiwan’s 2024 election (U.S. Department of State, 2024); AI-crafted memes and manipulated images promoting anti-US and anti-Japan messages (Meta, 2023); Coordination through bot networks to amplify all of the above (Stanford Internet Observatory, 2024). This campaign exemplifies the evolving sophistication of AI-driven disinformation. Instead of merely automating reposts, AI now creates original persuasive content – raising the ceiling of what bots can achieve in propaganda ecosystems. Detection efforts highlighted both the promise and the present limitations of deepfake forensics: while current-generation avatars showed tell-tale signs like robotic intonation and visual glitches, future iterations may become indistinguishable from authentic media. The Spamouflage case illustrates a turning point: disinformation actors now pair generative tools with automation to conduct scalable, persuasive, and potentially election-disrupting campaigns (Stanford Internet Observatory, 2024; Meta, 2023).

The broader implications of such AI-driven bot networks are far-reaching:

#### 4.5.5 Erosion of trust

As bots become more prevalent and harder to distinguish from real users, people grow more skeptical about online interactions and content. The phenomenon known as the “liar’s dividend” becomes a risk, where even truthful content is questioned because the public knows fakes are out there. When any opposing voice or inconvenient fact can be dismissed as “just a bot” or a deepfake, genuine democratic discourse suffers. For instance, authentic grassroots campaigns might struggle to gain credibility if observers suspect that social media support could be manufactured. UNESCO and Ipsos reported in 2024 that a majority of internet users worldwide feel they cannot be sure if what they see on social media is real or manipulated, indicating a general environment of distrust that is exacerbated by bot-driven disinformation (Ipsos and UNESCO, 2024).

#### 4.5.6 Manipulation of public opinion

AI-driven bots can manufacture consensus or at least the appearance of it. By making a particular viewpoint or hashtag trend, they can persuade neutral observers that “everyone is talking about this” or that a certain extreme position is more widely held than it truly is. This can attract media coverage (the press often reports on trending topics), further amplifying the desired narrative. For instance, during the “Reopen America” protests, the coordinated network made it seem as if there was a broad grassroots uprising against lockdowns, possibly influencing policymakers to consider lifting restrictions earlier than some health experts advised. Bots also engage in dogpiling – swarming individuals who voice opposing views with waves of criticism or harassment, sometimes driving them off the platform and silencing their perspective. In authoritarian contexts, regime-linked bots have been used to drown out dissenting hashtags by overwhelming them with pro-regime messages, effectively smothering opposition voices online.

#### 4.5.7 Information overload and “noise”

The ability of bots to produce content at a much higher volume than humans leads to an asymmetric flood of information. Studies have quantified this: one study found that bots can be 66 times more active than ordinary users, and in certain contentious discussions, bots made up nearly one-third of the content despite being a tiny fraction (under 1%) of participants (Rossetti and Zaman, 2023). This deluge can crowd out factual information. During breaking news events, for example, bot accounts might rapidly push misinformation or distracting content, complicating the job of journalists and fact-checkers to identify what’s true. For the average user, trying to sift through an avalanche of posts – some human, many automated – creates fatigue and confusion, which disinformation campaigns can exploit (people might start to believe a falsehood simply because they have seen it so many times in their feed, a familiarity effect).

#### 4.5.8 Amplification of low-credibility sources

Bots often serve to aggressively promote links from fringe websites or known propaganda outlets. Research shows that a significant portion of shares for content from “low-credibility” sources (those that regularly publish false or misleading info) can be attributed to bots. One study in 2024 indicated that about 33% of the top sharers of articles from dubious sites were likely automated accounts (George Washington University, 2024). This means bots can help such content leap into the mainstream conversation, whereas it might have languished in obscurity otherwise. By artificially boosting the hit counts and share counts of these articles, bots can even game platform algorithms that elevate content based on engagement metrics. This amplification is especially pernicious during elections or referendums, as false narratives from fringe outlets can get enough visibility to potentially sway undecided voters or reinforce polarized views.

#### 4.5.9 Undermining democratic processes

The presence of swarms of bots in political communication has raised alarms about electoral integrity. In the 2016 U.S. presidential election, analysts estimate that roughly 19% of tweets about the election came from bot accounts (Akamai, 2024). These accounts were found to be disproportionately pushing either extreme viewpoints or supportive messages for particular candidates, thus skewing the online discourse. In close races, the influence of bots – by driving agendas, altering perceptions of candidate popularity, or spreading disinformation about voting procedures – could even be outcome-determinative. More subtly, the knowledge that bots are interfering can reduce public confidence in election results; people might suspect the “voice of the people” was tainted by fake participants. All of this undermines the legitimacy of democratic outcomes and can contribute to unrest or refusal to accept results, as seen in various disputes where online disinfo played a role in fueling skepticism.

In response to the bot challenge, social media companies have tried to crack down by improving bot detection and removal. For example, Twitter (prior to its rebranding) routinely eliminated millions of suspected bot accounts every quarter and introduced rate limits to prevent excessive posting. However, the adversaries adapt as well – using techniques like time-shifting (to post during normal human hours) and content variation (to avoid obvious copy-paste patterns) to slip past filters. The cat-and-mouse game between platform moderators and bot creators is ongoing. On the policy side, some jurisdictions are considering or have passed laws requiring disclosure if content is produced by a bot (California enacted a B.O.T. act for certain commercial and political bots). But enforcement is tricky across borders.

One promising angle is the development of network analysis tools that look not just at individual accounts but at their behavior in aggregate, identifying telltale signs of coordinated campaigns. The 2024 Russian Meliorator network was discovered through such analysis, finding the connective tissue between accounts. Academic and industry researchers are increasingly collaborating to map out these networks (e.g., the Computational Propaganda Project at George Washington University cataloguing global bot operations).

Ultimately, tackling malicious bots may also require verification and authenticity standards on social media – such as optional verified identity for users (with trade-offs in terms of anonymity and privacy debates). If users can filter to see only verified humans, that could reduce bot impact, though it raises equity issues (not everyone has ID or wants to disclose it).

In conclusion, AI-driven bots are a cornerstone of contemporary disinformation efforts, capable of distorting online discourse at an unprecedented scale. They exploit the openness of social platforms and the psychological biases of users. Strengthening our defenses against them is critical to maintaining a functional digital public sphere.

### 4.6 Coordination at scale

The synergy of AI techniques across content creation, automation, and targeting enables large-scale coordinated disinformation campaigns that cut across platforms and national boundaries. Rather than isolated tactics, we now see integrated operations where AI monitors the information ecosystem, generates tailored content, and disseminates it through a web of channels for maximum impact. Key aspects and statistics illustrate how this coordination works and its effects:

Real-time social media analysis and strategy adaptation – disinformation campaigns are employing advanced AI-driven analytics to continuously scan social media trends, viral content, and user sentiment. By doing so, propagandists can identify emerging narratives or breaking news events to exploit. For example, if a natural disaster strikes, an AI system might quickly gauge public fears and then suggest disinformation angles (like conspiracies or blame narratives) to push. These systems can also detect when their false narratives are gaining or losing traction and adjust accordingly. This feedback loop allows for agile, adaptive tactics in almost real time. For instance, during an election, if a particular misleading talking point is not resonating, the AI may pivot to another line of attack that data shows is more compelling to the electorate.

Cross-platform botnet deployment – modern campaigns rarely limit themselves to a single platform. Using AI, malicious actors create interconnected bot networks that operate on multiple social media sites simultaneously. A coordinated approach might start a rumor on a fringe forum, amplify it via bots on Twitter, further propagate it through fake accounts on Facebook groups, and echo it in YouTube comments – all timed in concert. Such cross-platform coordination was observed in the earlier mentioned “Spamouflage” network linked to China, which spread content on Twitter, YouTube, Facebook, and even smaller platforms like Reddit and Tumblr, to maximize reach and create the appearance of a widespread movement. AI helps manage this complexity by centralizing control: operators can use dashboards to oversee their bot army’s activities across different sites, ensuring the message is consistent and ubiquitous. This also makes disinformation more resilient; if one platform cracks down, the narrative persists elsewhere and can even be reintroduced to the original platform via users who encountered it on a different site.

Natural language generation in multiple languages and styles – large language models enable disinformation agents to automatically generate content that is linguistically and culturally tailored. GPT-4, for example, can write persuasive texts not just in English but in dozens of languages, capturing nuances that a non-native speaker (or older machine translation tools) might miss. This capability means a single campaign can target audiences in different countries with messages calibrated to local context. It also means that AI-written propaganda can mimic the style of particular communities (e.g., using youth slang on one forum, formal terminology on another) to blend in. NewsGuard, a firm that tracks online misinformation, reported in 2024 that some propaganda sites were using AI to write articles that adapt to the normative tone of particular regions, making the misinformation harder to flag by readers who expect certain idioms or references. This multilingual, multi-style generation greatly increases the reach of disinformation and makes global narratives possible – such as coordinated COVID-19 misinformation that simultaneously targeted audiences in the US, Europe, and Asia with different culturally specific angles.

The impact of this large-scale coordination is evident in several statistics from recent studies:

The sheer volume and reach of AI-coordinated content is staggering. By one estimate, toxic misinformation spread via coordinated networks reaches millions of users daily and has affected political processes in over 50 countries (Johnson et al., 2024). This global penetration suggests that no region is immune; wherever social media use is prevalent, so too is the potential for AI-boosted disinformation to interfere with public discourse.

The prevalence of bots as part of internet traffic highlights the magnitude of the challenge. As of 2024, bots accounted for an estimated 42% of all web traffic, and importantly, about 65% of those bots were classified as malicious (engaged in activities like scraping, spamming, and disinformation) (Akamai, 2024). Legitimate bots (like search engine crawlers) are outnumbered by those with nefarious purposes. This indicates that a significant portion of online interactions or “users” at any given time might actually be automated agents. The internet landscape is thus partly artificial, and public opinion metrics gleaned from online data need to be interpreted with caution, knowing a large fraction could be bot activity.

In social media specifically, infiltration by fake accounts is massive. On X/Twitter, researchers in 2022 identified roughly 16.5 million accounts that were dedicated solely to pushing false information or spam narratives (Akamai, 2024). These are accounts that virtually contribute nothing genuine to discourse, only propaganda or scams. It shows how platforms can be flooded with inauthentic entities, which can confuse users (especially new or less savvy ones who may not suspect that a personable profile with a smiling face is a bot in disguise).

Bots’ ability to drive content is illustrated by specific events. During the first impeachment of U. S. President Donald Trump (late 2019 into 2020), it was found that bots generated 31% of the Twitter posts about the impeachment proceedings. These bots were often amplifying hyper-partisan or misleading narratives around the event (George Washington University, 2024). This level of automated involvement in a crucial national conversation raises questions about how public perception might have been swayed or distorted by inauthentic input.

Cross-platform connectivity – a study from George Washington University in 2024 revealed that disinformation operations routinely connect across an average of 23 different online platforms and services. This includes not just the big social media names, but also messaging apps, forums, comment sections of news sites, and more (George Washington University, 2024). By weaving threads through smaller niche sites (where moderation might be lax) into larger ones, campaigns can effectively launder the content – a false narrative might start in the dark corners of the web and then appear on Twitter cited as “seen on X forum,” then get into mainstream Facebook posts, etc., chaining its way to respectability.

The presence of unreliable AI-generated news sites further complicates the landscape. NewsGuard identified over 1,100 websites in 2023 that were essentially content mills using AI to produce news-like articles, often riddled with inaccuracies or sensationalism (NewsGuard, 2024). These sites, operating in multiple languages, churn out a high volume of clickbait and propaganda, and they monetize through ads. Because they produce so much content, their stories frequently surface in search engine results or social media shares. If someone searches a topical question, they might end up on one of these sites without realizing its dubious nature. The proliferation of such sites means that traditional cues people used to judge credibility (professional layout, volume of content, etc.) are no longer reliable – AI can provide all those, yet the information can be false.

Deepfake proliferation ties into coordination as well. MarketsandMarkets research in 2024 indicated online deepfake videos were doubling every six months since 2019, reaching at least 95,820 by 2023. Many of these are pornographic or frivolous, but a portion are used in coordinated influence or harassment campaigns (MarketsandMarkets, 2024). The accelerating trend suggests that deepfakes may become a more regular feature in disinformation operations, as tools to generate them become user-friendly and accessible.

Economic and confidence impacts – we have mentioned the $78 billion economic cost of misinformation in 2020, a figure that likely includes various productivity losses, scams, and mitigative expenses (World Economic Forum, 2020). Additionally, public confidence measurements, like the Ipsos/UNESCO finding that over 60% believe AI makes creating fake news easier, reflect a growing awareness that technology is amplifying falsehoods, which might paradoxically lead to greater skepticism of legitimate news as well (once again feeding into the “nothing is true” post-truth problem).

Bringing together states, private firms, and other actors highlights the key players in this coordinated disinformation landscape:

*State actors* (like Russian intelligence, Chinese state-affiliated groups, and Iran’s IRGC) use AI to scale up their information warfare and espionage activities. They typically have substantial resources and advanced tools (like Russia’s Meliorator). They also target not only foreign populations but sometimes domestic audiences to shape internal narratives (U.S. Department of Justice, 2024a, 2024b).

*Private “disinformation-for-hire” firms* have emerged, offering their services to anyone willing to pay. These companies operate in the shadows, providing clients with bot networks, fake news site infrastructure, deepfake generation, and more. Their existence lowers the barrier for smaller regimes or even non-state groups (like extremist organizations or wealthy individuals) to run sophisticated influence campaigns without needing an in-house capability (Carnegie Endowment for International Peace, 2024).

*Tech companies* inadvertently are enablers when their AI innovations are repurposed. Companies like OpenAI, Google, and Meta are continuously improving AI’s capabilities, which is beneficial for society in many ways, but also arms bad actors with better tools. There’s a tension here: do these companies have a responsibility to restrict access to their models or build in disinfo safeguards? They have taken some steps (like OpenAI’s content filters, or deepfake detection research by Facebook), yet the genie of general AI capability is out of the bottle and often open-source versions exist as well.

*Media organizations and civil society* are trying to respond by fact-checking, raising awareness, and creating initiatives like the Journalism Trust Initiative or deepfake databases. But they often play catch-up to the rapidly evolving tactics.

In face of this level of coordination, international cooperation and robust multi-stakeholder responses are critical. Already, collaborations like the NATO StratCom Center and the EU’s East StratCom task force are sharing information on disinformation campaigns. Tech companies have formed alliances (e.g., the Global Internet Forum to Counter Terrorism, which could serve as a model for disinformation). But a more comprehensive global strategy may be required, as recommended earlier in the policy section: something akin to a standing coalition or information defense pact.

As AI continues to advance (with looming prospects of even more powerful generative models, multimodal systems that combine text, video, and other media, and possibly AIs autonomously strategizing influence operations), the coordination and scale of disinformation could increase further. However, that same AI can be harnessed on the defensive side. The next subsection examines how AI is used to detect and counter disinformation, which is the hopeful flip side of this challenging coin.

### 4.7 AI in counter-disinformation efforts

While AI has supercharged the scale and sophistication of disinformation campaigns, it is also a powerful force for defending the integrity of the information space. Governments, technology firms, and civil society are increasingly deploying AI-driven tools to detect, verify, and mitigate false content online. These systems are being used not only to trace nefarious actions but also to preempt emerging threats, creating a growing ecosystem of proactive digital defense. To ensure effective deployment, however, it is crucial to distinguish clearly between the underlying AI capabilities and the intent behind their use. The same technologies that generate synthetic content can, when responsibly applied, expose malicious campaigns, support verification, and empower users with reliable information.

#### 4.7.1 Detection of inauthentic content and accounts

Machine learning models have become essential in identifying patterns characteristic of fake news, deepfakes, and coordinated disinformation activity. For example, Meta (formerly Facebook) uses AI-based systems to scan user content for known hoaxes or misleading claims, cross-referencing with fact-checking databases (Meta, 2023). Computer vision and audio analysis algorithms can detect manipulated videos or synthetic speech by examining inconsistencies in facial expressions or acoustic signatures. Initiatives such as the Deepfake Detection Challenge, organized in 2020, helped develop state-of-the-art classifiers that remain foundational in today’s detection workflows (Dolhansky et al., 2020).

#### 4.7.2 Network pattern analysis

Beyond content, AI is critical in identifying coordinated inauthentic behavior. Social media platforms apply graph-based algorithms to detect clusters of accounts that share anomalous characteristics – such as newly created profiles with AI-generated avatars engaging in synchronized posting. This methodology has proven effective in uncovering large-scale influence operations, as reported by Meta’s Coordinated Inauthentic Behavior reports.

#### 4.7.3 Automated fact-checking and stance detection

Natural Language Processing (NLP) enables the real-time extraction and assessment of claims within text. Tools such as Full Fact’s AI platform scan news and live transcripts for fact-checkable statements, aligning them with verified information (Full Fact, 2022). Similarly, stance detection algorithms analyze a document’s agreement or contradiction with known facts, flagging likely misinformation even before human review.

#### 4.7.4 Image and metadata verification

Reverse image search tools like TinEye or Google Fact Check Explorer employ AI to uncover the original context of repurposed or deceptive images. More advanced models analyze visual inconsistencies such as lighting mismatches, noise patterns, or shadow anomalies, allowing investigators to detect subtle manipulations in photographs shared across social platforms.

#### 4.7.5 Content moderation at scale

Platforms increasingly rely on AI to enforce content moderation rules at a speed and volume that human teams cannot match. For instance, during the COVID-19 pandemic, Facebook used AI to detect and remove or label over 20 million pieces of misinformation in a single quarter (Facebook Transparency Report, 2021). These models operate based on pre-defined misinformation heuristics, keyword analysis, and community flagging behaviors.

#### 4.7.6 Human-AI collaboration for monitoring

AI supports human analysts by filtering vast datasets to identify emerging narratives, disinformation trends, or suspicious user behavior in fringe forums and encrypted chats. This hybrid approach enables analysts to focus on high-impact threats, supported by AI-processed evidence of their reach, sentiment, and virality trajectories.

#### 4.7.7 Attribution and forensics

AI-powered systems analyze behavioral, linguistic, and infrastructural signatures across campaigns to attribute them to coordinated actors. One notable case is the attribution of the “Ghostwriter” campaign targeting European audiences, where linguistic forensics and shared infrastructure revealed a common origin behind dispersed incidents (Ben-Natan et al., 2021). These findings aid enforcement actions, including sanctions or indictments.

#### 4.7.8 User-facing verification tools

AI applications are also being developed for end-users in the form of browser extensions and mobile apps that offer real-time credibility assessments of online content. These systems might alert users to suspicious sources, highlight inconsistencies, or provide automated summaries with reliability indicators. Although still nascent, such tools mirror spam filters in email systems and can help cultivate critical media literacy at scale.

#### 4.7.9 Training and resilience via simulation

Gamified applications like “Bad News” and “Harmony Square” use AI to simulate disinformation environments and help users build psychological immunity to manipulative narratives. These tools adapt to user behavior, using reinforcement learning to personalize challenges and enhance learning effectiveness (Roozenbeek and van der Linden, 2019).

#### 4.7.10 Causal framework for AI misuse and defense

Across these examples, a clear causal pathway emerges: neutral technological capability (e.g., generative or predictive AI) → operationalization by malicious actors (e.g., coordinated inauthentic behavior) → measurable social harm (e.g., vaccine hesitancy, electoral interference). Importantly, each stage also provides a leverage point for ethical counter-action. The very tools exploited to scale disinformation – such as generative models or bot frameworks – can be repurposed for detection, simulation, and remediation. For instance, adversarial testing using synthetic bots helps platforms stress-test moderation filters, while generative models are used to train classifiers to recognize manipulated content.

## 5 Conclusion

In the evolving contest between disinformation and truth, AI is both a weapon and a shield. Its deployment must be grounded in an understanding that harm stems not from the existence of a tool, but from its application and the ecosystem of incentives surrounding it. Therefore, any policy framework must prioritize intent-based regulation, investment in cross-sector R&D, and transparency-by-design. As the information environment grows more complex, building a layered, adaptive, and evidence-based response that leverages the full spectrum of AI capabilities is the only viable path forward. This review underscores that safeguarding our digital public sphere is not a matter of banning technology, but of channeling it toward resilience, accountability, and collective intelligence.
